# Genomic characterization of carbapenemase-producing *Enterobacterales* from wastewater reveals the convergence of KPC-2 and GES-16 in Brazil

**DOI:** 10.1007/s10661-026-15867-0

**Published:** 2026-08-31

**Authors:** João Pedro Rueda Furlan, Giovanna Carrasco Bueno, Rubens Renato Sousa-Carmo, Renan Lourenço Oliveira Silva, Mikaela Renata Funada Barbosa, Maria Ines Zanoli Sato, Nilton Lincopan, Sergio Schenkman

**Affiliations:** 1 Department of Pharmaceutical Sciences, Health Sciences Center, Federal University of Paraíba, João Pessoa, Paraíba Brazil; 2 Escola Paulista de Medicina, Universidade Federal de São Paulo, São Paulo, Brazil; 3 Antimicrobial Resistance Institute of São Paulo (ARIES), São Paulo, Brazil; 4 Department of Microbiology, Instituto de Ciências Biomédicas, University of São Paulo, São Paulo, Brazil; 5 Department of Environmental Analysis, Environmental Company of São Paulo State (CETESB), São Paulo, Brazil; 6 Department of Clinical Analysis, Faculty of Pharmacy, University of São Paulo, São Paulo, Brazil

**Keywords:** Antimicrobial resistance, Carbapenemase-producing *Enterobacterales*, Whole-genome sequencing, Mobile genetic elements, South America

## Abstract

Carbapenemase-producing *Enterobacterales* (CPE) pose a significant public health concern due to the limited therapeutic options and increasing dissemination outside clinical settings. Wastewater treatment plants (WWTPs) have been proposed as relevant environmental reservoirs for antimicrobial-resistant bacteria and mobile genetic elements. In this study, we performed genomic characterization of CPE strains recovered from raw wastewater samples from the influents of different WWTPs. Antimicrobial susceptibility testing revealed multidrug resistance, including coresistance to carbapenems and polymyxins, among *Klebsiella pneumoniae*, *Enterobacter asburiae*, and *Enterobacter kobei* strains. Whole-genome sequencing identified the convergence of the *bla*_KPC-2_ and *bla*_GES-16_ genes, as well as the presence of *bla*_GES-5_ in *E. kobei* sequence type (ST) 540 strains. Moreover, the *bla*_KPC-2_ gene was detected in *K. pneumoniae* strains belonging to high-risk clones ST11 (capsular types KL64 and KL15) and ST307 (capsular type KL102), and *E. asburiae* ST384. The *bla*_GES-5_ and *bla*_GES-16_ genes were associated with class 1 integrons, while the *bla*_KPC-2_ gene was embedded within transposons (Tn*4401a*, Tn*4401i*, and Tn*3*-like) and insertion sequences (IS*Kpn27* and IS*Kpn6*). Notably, genomic analyses and literature review demonstrated that the *bla*_GES-16_ gene remains unique to Brazil. The putative pathogenic potential of carbapenem-resistant *K. pneumoniae* ST11 was also assessed. These findings support the environmental circulation of clinically relevant *Enterobacterales* genotypes and emphasize the potential role of WWTPs as conduits for CPE dissemination, if not adequately operated. Therefore, genomic surveillance in extra-hospital settings may contribute to a better understanding of antimicrobial resistance ecology and inform One Health mitigation strategies.

**Supplementary Information:**

The online version contains supplementary material available at https://doi.org/10.1007/s10661-026-15867-0.

## Introduction

Carbapenem-resistant *Enterobacterales* (CRE) have emerged as critical threats to global public health due to their association with limited therapeutic options, high morbidity, and mortality (Gürbüz & Gencer, 2024). Accordingly, the World Health Organization has classified CRE among the critical priority pathogens (Sati et al., 2025). Among CRE, carbapenem-resistant *Klebsiella pneumoniae* has become one of the most prominent pathogens causing nosocomial infections worldwide, with *K. pneumoniae* accounting for a large proportion of carbapenem-resistant bloodstream and healthcare-associated bloodstream infections (BSIs) across multiple regions, representing 14.2% of CRE in Brazilian BSIs (Antochevis et al., 2025).

In extra-hospital settings, wastewater treatment plants (WWTPs) are increasingly recognized as critical environmental reservoirs and routes for antimicrobial resistance (AMR) dissemination (La Rosa et al., 2025). Wastewater often contains a mixture of bacteria, xenogenetic pollutants, and selection compounds, creating favorable conditions for horizontal gene transfer and the persistence of antimicrobial-resistant species in the environmental sector (Karkman et al., 2018; Mutuku et al., 2022). Wastewater surveillance of AMR by genomic analysis offers a powerful approach to monitor critical priority pathogens. By analyzing wastewater, it becomes feasible to detect early signals of pathogen circulation, track the appearance of novel or regionally emerging species, clones, and antimicrobial resistance genes (ARGs), and observe unusual combinations of AMR and virulence traits (Sousa-Carmo & Lincopan, 2025).

From a One Health perspective, the rapid dissemination of carbapenemase-producing *Enterobacterales* (CPE) is facilitated by the mobilization of carbapenemase genes via mobile genetic elements (MGEs), often coupled with clonal expansion (Ragheb et al., 2022). The role of MGEs in disseminating carbapenemase genes has been documented across South America, demonstrating how these elements drive intra- and interspecies transmission dynamics and exacerbate the AMR crisis (Reyes et al., 2020). Furthermore, the presence of class 1 integrons in WWTPs remains a reliable signal of AMR burden and dissemination potential (Tavares et al., 2024). Therefore, this study aimed to characterize CPE strains recovered from wastewater samples from WWTPs in Brazil and understand the genetic environments surrounding the carbapenemase genes.

## Materials and methods

### Sampling sites and bacterial obtaining

Between April and May 2024, raw wastewater samples (500 mL) were collected from the influents of four WWTPs, denominated Ribeirão Preto (RP, 21°06′17.9″S 47°48′42.3″W), Riacho Grande (RG, 23°46′39.5″S 46°31′59.8″W), Parque Novo Mundo (PNM, 23°30′53.0″S 46°33′27.0″W), and Barueri (BA, 23°30′51.0″S 46°50′39.0″W), located in the state of São Paulo, Brazil. The samples were filtered using nitrocellulose membranes with 0.45-µm pore size (Merck Millipore Ltd., Ireland), and the membranes were added to test tubes with 5 mL of Brain Heart Infusion Broth (BHI, Kasvi, Spain), which were incubated at 37 ± 2 °C for 24 h. Then, 50 µL of each culture was seeded on plates of BBL™ CHROMagar™ Orientation plates (BD, France) with 4 mg/L of meropenem and vancomycin, and the plates were incubated at 37 ± 2 °C for 24 h. Finally, macroscopically different colonies were selected and identified using matrix-assisted laser desorption ionization–time of flight mass spectrometry (MALDI-TOF MS) (Bruker Daltonics, Germany). For *K. pneumoniae* strains, the hypermucoviscous phenotype was evaluated by the Sting test (Shon et al., 2013).

### Antimicrobial susceptibility testing and phenotypic identification of carbapenemases

The disk diffusion method was performed to determine the antimicrobial susceptibility to meropenem (MPM, 10 µg), imipenem (IPM, 10 µg), aztreonam (ATM, 30 µg), ceftazidime + avibactam (CZA, 30/20 µg), ceftazidime (CAZ, 30 µg), ceftriaxone (CRO, 30 µg), cefepime (CPM, 30 µg), cefoxitin (CFO, 30 µg), ampicillin + sulbactam (APS, 10/10 µg), amoxicillin + clavulanic acid (AMC, 20/10 µg), trimethoprim + sulfamethoxazole (SXT, 1.25/23.75 µg), ciprofloxacin (CIP, 5 µg), tetracycline (TET, 30 µg), minocycline (MIN, 30 µg), gentamicin (GEN, 10 µg), amikacin (AMI, 30 µg), chloramphenicol (CHL, 30 µg), and nitrofurantoin (NIT, 300 µg) (CECON, Brazil). Additionally, the broth microdilution method was used to determine the minimum inhibitory concentration (MIC) for MPM, colistin (COL), and tigecycline (TGC) (CLSI, M100™, 34th ed., 2024; EUCAST, v.14.0, 2024; EUCAST, 2006). Phenotypic carbapenemase production was assessed by inhibitor-based methods (Pournaras et al., 2010).

### Whole-genome sequencing and bioinformatic analyzes

Genomic DNA was extracted using the PureLink™ Genomic DNA Mini Kit (Thermo Fisher Scientific, USA), and short-read sequencing was performed in the Illumina HiSeq platform (Illumina Inc., USA). The draft genome was de novo assembled by SPAdes v.3.15.2 (https://github.com/ablab/spades), and its quality was checked using CheckM v.1.1.6 (https://github.com/Ecogenomics/CheckM) (Supplementary Table S1). The bacterial identification at the species level was assessed by average nucleotide identity (ANI) and digital DNA-DNA hybridization (dDDH) analyses (Meier-Kolthoff et al., 2022; Richter et al., 2016). Resistome (acquired and intrinsic ARGs, point mutations, and biocide and metal tolerance genes), plasmid replicons, mobilome (transposons, integrons, and insertion sequences), virulome, and molecular typing (multilocus sequence typing and surface polysaccharide typing) were determined using tools available at the Center for Genomic Epidemiology (https://www.genomicepidemiology.org/), Microbial Ecology Group (https://www.meglab.org/), ISfinder (https://www-is.biotoul.fr/index.php), BLAST® (https://blast.ncbi.nlm.nih.gov/Blast.cgi), BIGSdb-Pasteur (https://bigsdb.pasteur.fr/), VFDB (https://www.mgc.ac.cn/VFs/), and Pathogenwatch (https://pathogen.watch/). For colistin-resistant *K. pneumoniae* strains, mutations in polymyxin resistance determinants were searched and analyzed as previously described by Furlan et al. (2023).

### Data availability

The nucleotide sequences of all CPE strains used in this study are available in the NCBI database under the BioProject number PRJNA1216268.

## Results and discussion

### CRE and CPE strains

Twelve CRE bacterial strains were isolated in the WTTPs studied. Species identification revealed *Enterobacter asburiae* (*n* = 1), *Enterobacter kobei* (*n* = 2), and *K. pneumoniae* (*n* = 9). Identification was supported by MALDI-TOF MS (score > 2.0), and it was confirmed genomically by ANI (> 95%) and dDDH (> 70%) (Table 1). All strains displayed multidrug resistance, with resistance to carbapenems, and harbored genes encoding serine carbapenemases. MIC values for meropenem were > 64 mg/L. Colistin MICs ranged from 1 to > 64 mg/L, with 75% (9/12) of strains classified as colistin-resistant (Table 1). In contrast, all strains were susceptible to CZA and TGC (MIC ≤ 2 mg/L). Phenotypic screening revealed carbapenemase activity in all strains, with phenylboronic acid inhibition suggesting serine carbapenemases (Supplementary Table S2). Consistently, the carbapenemase genes *bla*_KPC-2_, *bla*_GES-5_, and *bla*_GES-16_ were identified, with *bla*_KPC-2_ predominating (11/12; 91.6%). Notably, *E. kobei* strain M1 coharbored *bla*_KPC-2_ and *bla*_GES-16_ genes (Table 2).

**Table 1:** Isolation source, species, and AMR profiles of the bacterial strains used in this study

Isolation source^a^	Strain	Species	AMR profile (MIC mg/L)^b^
RP-A	M1	*E. kobei*	CRO, CAZ, CPM, ATM, MPM (> 64), IPM, CIP, GEN, AMI, NIT
RP-A	M2	*K. pneumoniae*	AMC, APS, CFO, CRO, CAZ, CPM, ATM, MPM (> 64), IPM, CIP, GEN, AMI, TET, MIN, CHL, SXT, NIT, COL (> 64)
RP-A	M5	*K. pneumoniae*	AMC, APS, CFO, CRO, CAZ, CPM, ATM, MPM (> 64), IPM, CIP, GEN, MIN, SXT, NIT, COL (4)
RG-A	M9	*E. asburiae*	CRO, CAZ, CPM, ATM, MPM (> 64), IPM, CIP, AMI, MIN, NIT, COL (> 64)
PNM-A	M15	*K. pneumoniae*	AMC, APS, CFO, CRO, CAZ, CPM, ATM, MPM (> 64), IPM, CIP, GEN, TET, MIN, SXT, NIT, COL (4)
PNM-A	M37	*E. kobei*	CRO, CAZ, CPM, MPM (> 64), IPM, CIP, AMI, TET, MIN, CHL, FOS, COL (> 64)
PNM-B	M17	*K. pneumoniae*	AMC, APS, CFO, CRO, CAZ, CPM, ATM, MPM (> 64), IPM, CIP, GEN, AMI, TET, MIN, CHL, SXT, NIT, COL (> 64)
PNM-B	M36	*K. pneumoniae*	AMC, APS, CFO, CRO, CAZ, CPM, ATM, MPM (> 64), IPM, CIP, TET, MIN, CHL, SXT, NIT, COL (> 64)
BA-A	M30	*K. pneumoniae*	AMC, APS, CFO, CRO, CAZ, CPM, ATM, MPM (> 64), IPM, CIP, GEN, AMI, TET, MIN, SXT, NIT
BA-A	M33	*K. pneumoniae*	AMC, APS, CFO, CRO, CAZ, CPM, ATM, MPM (> 64), IPM, CIP, GEN, AMI, TET, MIN, SXT, NIT
BA-B	M32	*K. pneumoniae*	CRO, CAZ, CPM, ATM, MPM (> 64), IPM, CIP, TET, MIN, SXT, NIT, COL (8)
BA-B	M35	*K. pneumoniae*	AMC, APS, CFO, CRO, CAZ, CPM, ATM, MPM (> 64), IPM, CIP, MIN, CHL, SXT, COL (> 64)

^a^Collection date: RP-A and RG-A (04/17/2024); PNM-A (04/25/2024); BA-A (05/14/2024); PNM-B and BA-B (05/16/2024)

^b^*Enterobacter cloacae* complex has intrinsic resistance to AMC, APS, and CFO tested

**Table 2:** Genomic characteristics of CPE strains from this study

Strain	Typing^d^		Antimicrobial resistance gene^e^	Virulence determinant^f^	Plasmid replicon
	ST	Serotyping			
M1^a^	520	na	*bla*_KPC-2_, *bla*_GES-16_, *bla*_TLA-like_, *bla*_ACT-102_, *aac(6′)-Ib3*, *tet(C)*, *msr(E)*, *mph(E)*	nd	IncFIB(K), IncP6, Col(pHAD28), Col(IRGK)
M2^b^	11	O1/O2v1:KL64	*bla*_KPC-2_, *bla*_CTX-M-2_, *bla*_OXA-2_, *bla*_TEM-1B_, *bla*_SHV-11_, *oqxA*, *oqxB*, *rmtB*, *aac(3)-IId*, *aac(6′)-Ib3*, *aph(3″)-Ib*, *aph(6)-Id*, *tet(G)*, *sul1*, *sul2*, *catA1*, *mph(A)*, *erm(42)*, *fosA*^KP^	*mrk* gene cluster, ICE*Kp10*, *clb* 3	IncFII(K), IncFIB(pQil), IncFIB(K), ColRNAI
M5^b^	307	O1/O2v2:KL102	*bla*_KPC-2_, *bla*_CTX-M-15_, *bla*_OXA-1_, *bla*_TEM-1B_, *bla*_SHV-28_, *qnrB1*, *oqxA*, *oqxB*, *aac(6′)-Ib-cr*, *aph(3″)-Ib*, *aph(6)-Id*, *aac(3)-IIa*, *sul2*, *dfrA14*, *catB3*, *mph(A)*, *fosA*^KP^	*mrk* gene cluster	IncFII(K), IncFIB(K), IncC3
M9^c^	384	na	*bla*_KPC-2_, *bla*_OXA-9_, *bla*_ACT-34_, *bla*_TEM-1A_, *aac(6′)-Ib*, *aph(3′)-VIa*, *aadA1*	*fimA*, *fimC*, *fimD*, *fimI*, *fimZ*, *fepA*, *entB*, *entE*, *entS*	IncFII(Yp), IncFII(SARC14), IncFIA(HI1), IncFIB(K)
M15^b^	307	O1/O2v2:KL102	*bla*_KPC-2_, *bla*_CTX-M-15,_ *bla*_OXA-1_, *bla*_TEM-1B_, *bla*_SHV-28_, *qnrB1*, *oqxA*, *oqxB*, *aac(6′)-Ib-cr*, *aac(3)-IIa*, *aph(6)-Id*, *aph(3″)-Ib*, *catB3*, *sul2*, *dfrA14*, *mph(A)*, *fosA*^KP^	*mrk* gene cluster	IncFII(K), IncFIB(K), IncC3
M37^a^	520	na	*bla*_GES-5_, *bla*_OXA-10_, *bla*_ACT-102_, *qnrVC4*, *aph(3′)-*VIa, *aac(6′)-Ib3*, *aadA1*, *cmlA5*, *dfrA14*, *msr(E)*, *mph(E)*, *fosA*^KP^	nd	IncFIB(K), IncQ1, IncP6
M17^b^	307	O1/O2v2:KL102	*bla*_KPC-2_, *bla*_CTX-M-15_, *bla*_OXA-1_, *bla*_TEM-1B_, *bla*_SHV-28_, *qnrB1*, *oqxA*, *oqxB*, *aac(6′)-Ib-cr*, *aac(3)-IIa*, *aph(6)-Id*, *aph(3″)-Ib*, *sul2*, *dfrA14*, *catB3*, *mph(A)*, *fosA*^KP^	*mrk* gene cluster	IncFII(K), IncFIB(K), IncC3
M36^b^	11	O1/O2v1:KL64	*bla*_KPC-2_, *bla*_SHV-11_, *aac(6′)-Ib3*, *oqxA*, *oqxB*, *tet(A)*, *sul1*, *sul2*, *dfrA30*, *mph(A)*, *fosA*^KP^	*mrk* gene cluster, ICE*Kp10*, *clb* 3	IncFIB(K), IncFIB(K), IncFIB(pQil), IncN, ColRNAI, Col440ll
M30^b^	11	O4:KL15	*bla*_KPC-2_, *bla*_CTX-M-15_, *bla*_LAP-2_, *bla*_OXA-9_, *bla*_OXA-1_, *bla*_TEM-1A_, *bla*_SHV-11_, *aph(3′)-Ia*, *qnrS1*, *oqxA*, *oqxB*, *aac(6′)-Ib-cr*, *aac(3)-IIa*, *aadA2*, *tet(A)*, *sul1*, *sul2*, *dfrA12*, *catB3*, *mph(A)*, *fosA*^KP^	*mrk* gene cluster, ICE*Kp3*	IncFII(K), IncFIB(K), IncFIB(pQil), IncC, IncN-pST15, ColRNAl, Col440l
M33^b^	11	O1/O2v1:KL64	*bla*_KPC-2_, *bla*_CTX-M-2_, *bla*_OXA-2_, *bla*_TEM-1B_, *bla*_SHV-11_, *oqxA*, *oqxB*, *rmtB*, *aac(3)-IIa*, *aac(6′)-Ib3*, *aph(6)-Id*, *aph(3″)-Ib*, *tet(G)*, *sul1*, *sul2*, *catA1*, *erm(42)*, *mph(A)*, *fosA*^KP^	*mrk* gene cluster, ICE*Kp10*, *clb* 3	IncFII(K), IncFIB(K), IncFIB(pQil), ColRNAI
M32^b^	11	O4:KL15	*bla*_KPC-2_, *bla*_CTX-M-15_, *bla*_LAP-2_, *bla*_OXA-9_, *bla*_OXA-1_, *bla*_TEM-1A_, *bla*_SHV-11_, *aph(3′)-Ia*, *qnrS1*, *oqxA*, *oqxB*, *aac(6′)-Ib-cr*, *aac(3)-IIa*, *aadA2*, *tet(A)*, *sul1*, *sul2*, *dfrA12*, *catB3*, *mph(A)*, *fosA*^KP^	*mrk* gene cluster, ICE*Kp3*	IncFII(K), IncFIB(K), IncFIB(pQil), IncC, IncN-pST15, ColRNAl, Col440l
M35^b^	307	O1/O2v2:KL102	*bla*_KPC-2_, *bla*_CTX-M-15_, *bla*_OXA-1_, *bla*_TEM-1B_, *bla*_SHV-28_, *qnrB1*, *oqxA*, *oqxB*, *aac(6′)-Ib-cr*, *aac(3)-IIa*, *aph(3″)-Ib*, *aph(6)-Id*, *sul2*, *dfrA14*, *mph(A)*, *fosA*^KP^	*mrk* gene cluster	IncFII(K), IncFIB(K), IncC3

^a^*E. kobei*

^b^*K. pneumoniae*

^c^*E. asburiae*

^d^ST sequence type, na not applied

^e^Truncated ARGs were not listed

^f^nd not detected

The environmental spread of CRE and CPE, particularly through inadequately treated wastewater discharged from WWTPs, is a key factor driving AMR within the One Health perspective (Larsson & Flach, 2022). In Brazil, CPE strains have been reported in wastewater samples from WWTP effluents (Montenegro et al., 2023). Recently, *Citrobacter freundii* sequence type (ST) 522 and *K. pneumoniae* ST258 strains were detected as harboring novel *bla*_KPC-2_ allelic variants in the PNM site (Furlan et al., 2025a), while unusual species such as *Comamonas resistens* carrying the *bla*_GES-5_ gene (Furlan et al., 2025b) and *Serratia nevei* harboring the *bla*_NDM-1_ gene were identified in the RG site (Furlan et al., 2025c). These findings reinforce the role of WWTPs as environmental reservoirs and mixing points for clinically relevant AMR determinants, high-risk lineages, and unusual species (La Rosa et al., 2025).

### High-risk and international clones encompassing a wide range of genomic traits

*K. pneumoniae* strains belonged to recognized high-risk clones, namely, ST11 and ST307 (Table 2). ST11 was recovered on different WWTPs and collection dates, indicating a nonsporadic presence and multipoint environmental input, rather than a single isolated contamination event. In addition, the occurrence of two distinct capsular (KL) types (KL64 and KL15) within ST11 showed capsular diversity, a feature that may reflect ecological plasticity and the contribution of different source populations to the sewage network (Chen et al., 2023; Zhou et al., 2020). For ST307, although only one capsular type (KL102) was observed, its detection in more than one WWTP also supports the hypothesis of multiple introduction routes, consistent with this clone’s strong healthcare association and its ability to spread through urban wastewater systems (Peirano et al., 2020; Wyres et al., 2019). These high-risk clones are globally successful in causing healthcare-associated infections and frequently harbor carbapenemase genes, with ST11-KL64 typically linked to highly transmissible, virulent, and carbapenemase-producing sublineages, whereas ST11-KL15 has been associated with more heterogeneous epidemiological backgrounds, often reflecting broader environmental or community dissemination (Chen et al., 2025; Shi et al., 2024; Wang et al., 2023).

*E. kobei* strains belonged to international clone ST520. This clone has been previously reported as carbapenem-resistant and recovered from coastal waters from Brazil (Sellera et al., 2017) and clinical samples from China, India, and Spain (Halder et al., 2025; Peirano et al., 2018; Wang et al., 2017; Zhao et al., 2020), showing its occurrence across geographically distant regions. Conversely, the *E. asburiae* strain M9 belonged to ST384, for which no previous reports were found, indicating that this lineage may be rare or undercharacterized. Although its epidemiological relevance remains unclear, the detection of ST384 in wastewater underscores how environmental genomic surveillance can reveal previously unreported *Enterobacter* species diversity.

In addition to serine carbapenemase genes, all strains harbored acquired and intrinsic genes mainly related to resistance to β-lactams, fluoroquinolones, aminoglycosides, tetracyclines, sulfonamides, and phenicols (Table 2). Accordingly, genes encoding extended-spectrum β-lactamases (*bla*_CTX-M-2_ and *bla*_CTX-M-15_) and a 16S rRNA methyltransferase (*rmtB*) stand out, since they confer resistance to clinically important antimicrobials, such as third-generation cephalosporins (e.g., ceftriaxone) and aminoglycosides (e.g., amikacin), respectively. All fluoroquinolone-resistant *K. pneumoniae* strains presented mutations in the quinolone resistance-determining regions of GyrA (S83I) and ParC (S80I) (Supplementary Table S3), whereas multiple mutations were detected in polymyxin resistance determinants among colistin-resistant *K. pneumoniae* strains, including the disruption of *mgrB* by an IS*Kpn14* element and a deleterious R256G substitution in PmrB (Supplementary Table S4). These results corroborate the phenotype-genotype concordance widely reported in *Enterobacterales*, where the presence of known ARGs, point mutations, and insertion/disruption events detected by genomics generally predicts the AMR phenotypes observed in susceptibility testing (Li et al., 2024; Vanstokstraeten et al., 2023). Moreover, genes associated with tolerance to biocides (quaternary ammonium compounds) and metals (arsenic, copper, mercury, and silver) were also identified (Supplementary Table S5).

The putative pathogenic potential of *K. pneumoniae* and *Enterobacter* spp. was assessed based on virulence determinants. All *K. pneumoniae* strains carried the *mrk* gene cluster encoding type 3 fimbriae, which are associated with adhesion, colonization, and biofilm formation (Liu et al., 2024). Additionally, the yersiniabactin siderophore locus embedded in integrative conjugative elements ICE*Kp* was detected, corresponding to ICE*Kp3* and ICE*Kp10* variants in ST11-KL15 and ST11-KL64 strains, respectively. This virulence mechanism facilitates iron acquisition, immune evasion, and oxidative stress resistance, contributing to increased pathogenicity (Tan et al., 2024). Notably, the ST11-KL64 strain also harbored the colibactin gene cluster, specifically the *clb* 3 variant, which encodes a genotoxin linked to DNA damage, epithelial barrier disruption, and persistence during infection (Lan et al., 2019). *K. pneumoniae* ST307-KL102 strains presented the hypermucoviscosity phenotype, demonstrating increased capsule production that enhances resistance to host immune responses (Heiden et al., 2020).

Conversely, the virulence determinants of *Enterobacter* species remain rather unclear (Davin-Regli et al., 2019). *E. asburiae* strain M9 carried determinants associated with adhesion (*fim* genes) and iron acquisition (*fepA*, *entB*, *entE*, and *entS*) (Table 2), which are characteristic of opportunistic *Enterobacterales* and support host colonization rather than hypervirulence (Amaretti et al., 2020; Azam et al., 2023). In *E. kobei*, no classical or experimentally validated virulence determinants were detected (Table 2). This pattern is consistent with the opportunistic lifestyle of *Enterobacter* species, in which pathogenicity largely depends on basic fitness traits and host susceptibility, rather than on specialized virulence systems typical of hypervirulent *Enterobacterales*. In contrast, the detection of putative hypervirulent, hypermucoviscous, and carbapenem-resistant *K. pneumoniae* in WWTPs is particularly concerning, as it demonstrates the environmental circulation of high-risk clones harboring ARGs and virulence-associated features.

The isolated CPE strains also carried several plasmid replicons, particularly IncF-type, which are well recognized for disseminating AMR and virulence determinants among *Enterobacterales* (Rozwandowicz et al., 2018). Furthermore, IncN-pST15 and IncP6 plasmid replicons were also identified. These plasmids have been described as carrying the *bla*_KPC-2_ gene in *K. pneumoniae* and *Escherichia coli* strains from surface water and agricultural soil in southeastern Brazil (Furlan et al., 2023, 2024), reinforcing the regional environmental dissemination of carbapenemase genes. However, due to methodological constraints associated with short-read sequencing, it was not possible to fully resolve and assemble these plasmids into complete contigs, preventing a more detailed reconstruction of their genomic architecture and cargo (Arredondo-Alonso et al., 2017).

### Genetic contexts of serine carbapenemase genes

**Fig. 1:**
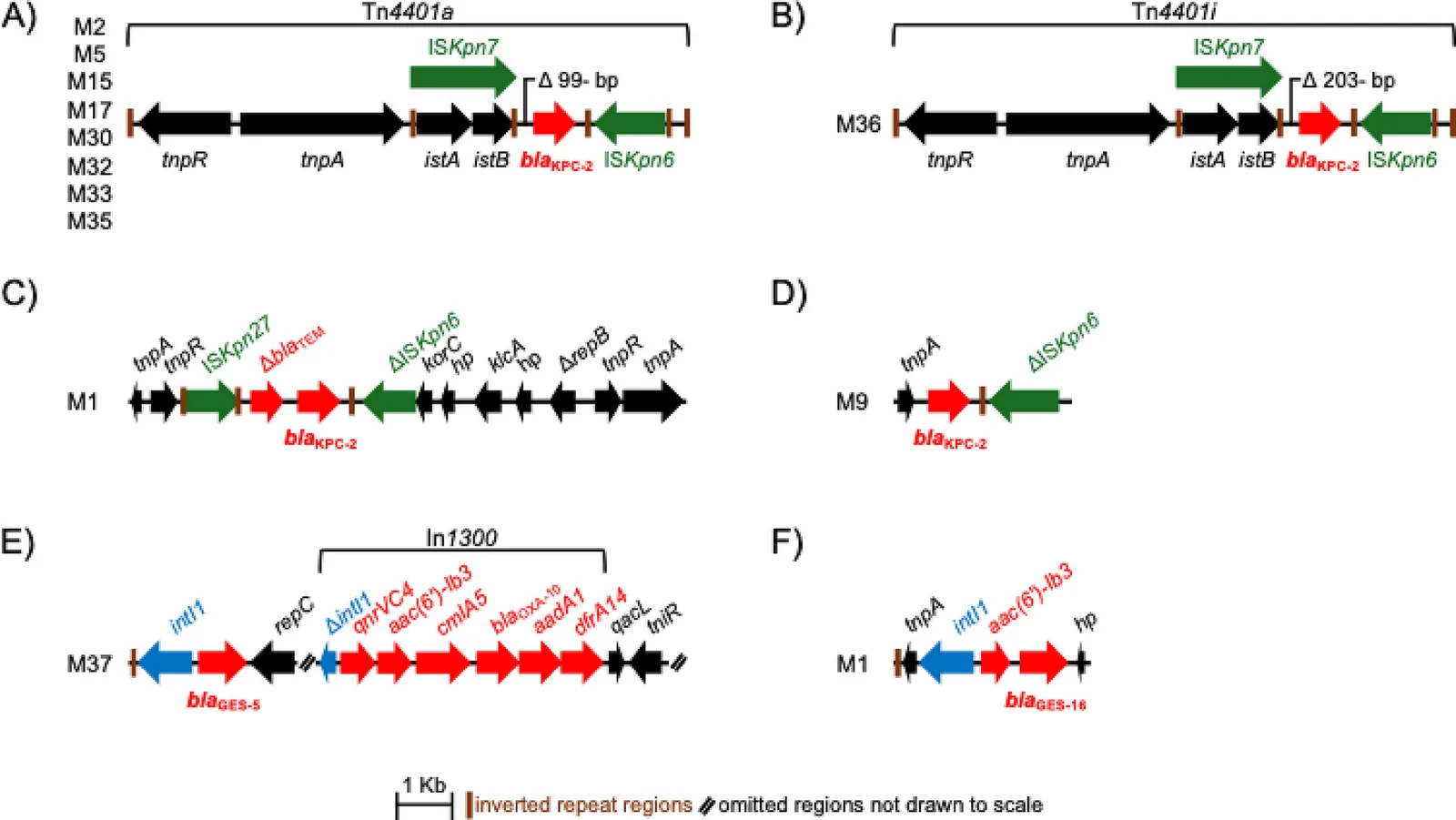
Schematic representation of the genetic contexts harboring genes encoding serine carbapenemases in the indicated isolates. **A** The *bla*_KPC-2_ gene is embedded in the Tn*4401a* transposon (GenBank: KT378596). **B** The *bla*_KPC-2_ gene embedded in the Tn*4401i* transposon (GenBank: MH047294). **C** The *bla*_KPC-2_ gene is associated with IS*Kpn27* and IS*Kpn6*. **D** The *bla*_KPC-2_ gene is associated with IS*Kpn6*. **E** Fragment harboring the *bla*_GES-5_ gene associated with class 1 integron integrase gene *intI1* and In*1300* integron (GenBank: CP022170). **F** The *bla*_GES-16_ gene is associated with the class 1 integron integrase gene *intI1*. Genes encoding serine carbapenemases are highlighted in bold. Hypothetical protein, hp

All *K. pneumoniae* strains, except the M36 strain, carried the *bla*_KPC-2_ gene embedded within the Tn*4401a* transposon (Fig. 1A). Conversely, the *bla*_KPC-2_ gene from the M36 strain was located within the Tn*4401i* isoform (Fig. 1B). Classical transposons, such as Tn*4401*, have been central to the global dissemination of *bla*_KPC-2_, particularly among hospital-associated *K. pneumoniae* lineages, but structural variability within Tn*4401* isoforms, especially Tn*4401a*, has been increasingly documented and linked to differences in mobility and epidemiological impacts (Naas et al., 2008; Reyes et al., 2020). Curiously, the uncommon Tn*4401i* isoform was reported in high-risk clones of *K. pneumoniae* ST11 and ST258 from clinical and environmental sectors of Brazil (Araújo et al., 2018; Rosa et al., 2024), but its epidemiological risk remains unclear, highlighting the need for continued surveillance.

In *E. kobei* strain M1 and *E. asburiae* strain M9, the *bla*_KPC-2_ gene was inserted into IS*Kpn27*-Δ*bla*_TEM_-*bla*_KPC-2_-ΔIS*Kpn6* (Fig. 1C) and *tnpA*-*bla*_KPC-2_-ΔIS*Kpn6* (Fig. 1D) genetic structures, respectively. These platforms also carried *tnpA* genes related to the Tn*3*-family transposon. Genetic contexts involving Tn*3*-associated transposases indicate that nonclassical mobilization routes, which may facilitate carbapenemase capture and transfer across diverse hosts, reflect the plasticity of resistance loci (Casacuberta & González, 2013; Partridge et al., 2018). Accordingly, these genetic platforms could represent early stages of mobilization pathways before stabilization in clinically dominant vehicles, underscoring their potential role in shaping emerging resistance dynamics.

The *bla*_GES-5_ (Fig. 1E) and *bla*_GES-16_ (Fig. 1F) genes identified in *E. kobei* strains M1 and M37 were surrounded by the class 1 integron integrase gene *intI1* with *attI* and *attC* regions. Furthermore, the In*1300* integron was identified in the same fragment of the *bla*_GES-5_ gene (Fig. 1E). The association of *bla*_GES_ allelic variants with class 1 integrons highlights a highly efficient mechanism of genetic mobilization, as these evolutionary platforms can capture, excise, and rearrange gene cassettes through the *intI1* integrase, thereby promoting the accumulation and transfer of clinically relevant ARGs (Gillings, 2014). Therefore, these MGE-mediated recruitments and spread are particularly concerning in anthropogenically impacted environments, ultimately bridging the clinical-environmental interface and enhancing the adaptive potential of the resistome (Gillings, 2017; Khedkar et al., 2022).

### Genomic context of the *bla*_GES-16 _gene

**Fig. 2:**
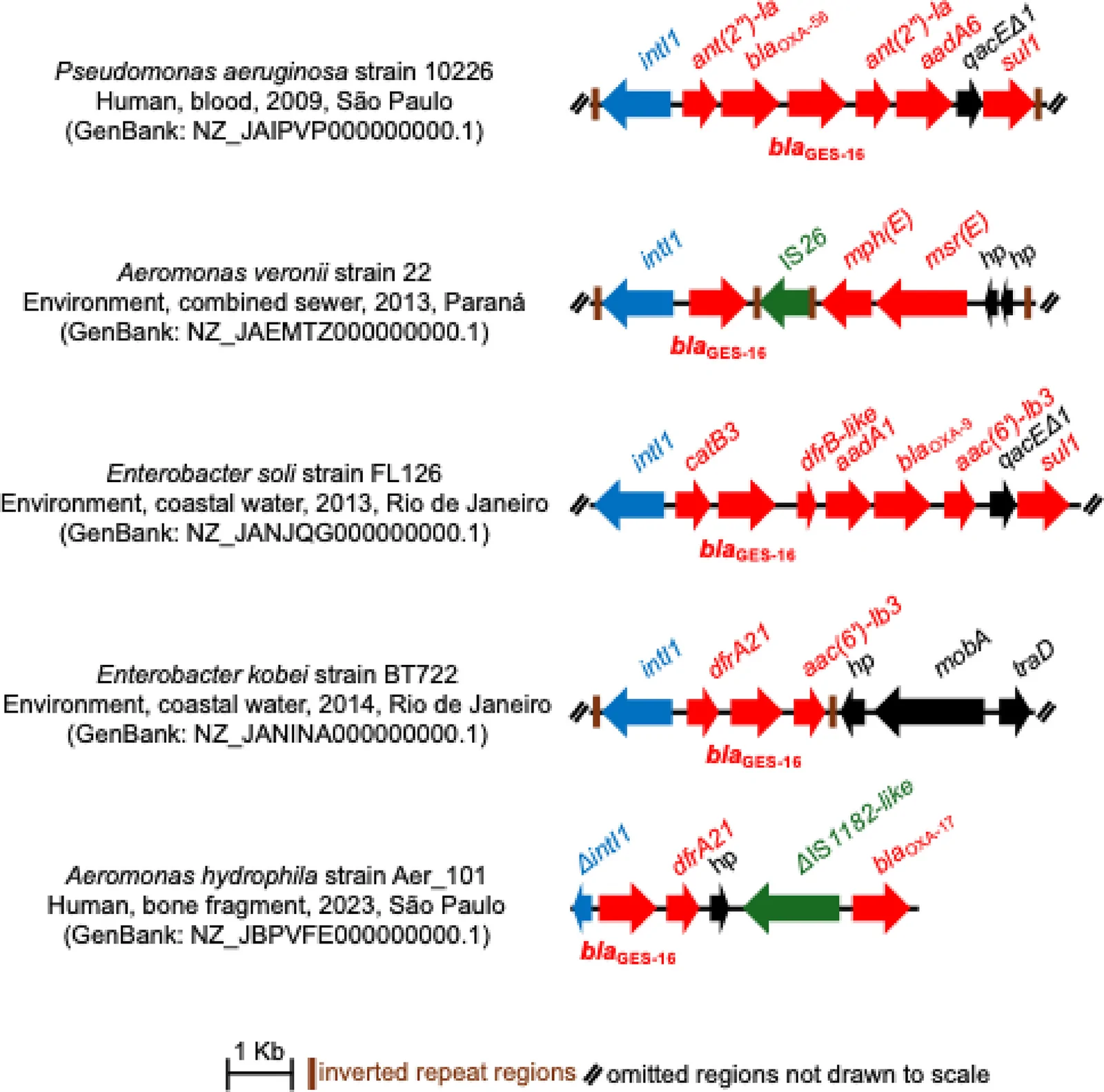
Schematic representation of integron-related sequences containing the *bla*_GES-16_ gene in Brazilian genomes. The *bla*_GES-16_ gene is highlighted in bold. Hypothetical protein, hp

GES-16 carbapenemase, encoded by the *bla*_GES-16_ gene, differs from the GES-5 carbapenemase by a single amino acid substitution (Gln38Glu). This carbapenemase was first described in clinical strains of *Serratia marcescens* from Rio de Janeiro, Brazil (Streling et al., 2018). Subsequently, *bla*_GES-16_ was also detected in *Aeromonas veronii* strains from hospital wastewater in Paraná (Conte et al., 2021, 2022). By using the NCBI’s genome resources on January 7, 2026, only five public genomes carrying *bla*_GES-16_ were identified, all originating from Brazil (Fig. 2), indicating that its known distribution remains geographically restricted. This gene was observed in clinical and environmental strains of *Aeromonas hydrophila* (ST508), *A. veronii* (ST886), *Enterobacter kobei* (ST32), *Enterobacter soli*, and *Pseudomonas aeruginosa* (ST3603) isolated from different Brazilian states (Fig. 2). Accordingly, the distribution of the *bla*_GES-16_ gene remains geographically restricted to Brazil. Additionally, GES-16-positive FL126, BT722, and Aer_101 genomes also carried the KPC-2 carbapenemase.

In the genomes studied, the *bla*_GES-16_ gene was identified on chromosome and plasmid contigs, which were inferred from nucleotide BLAST alignment. It was consistently associated with the class 1 integron integrase gene *intI1*, highlighting the role of the class 1 integrons in the maintenance of this gene. Moreover, ARGs, including the *bla*_OXA-17_ gene that encodes an extended-spectrum β-lactamase and insertion sequences, were also found surrounding the *bla*_GES-16_ gene (Figs. 1F and 2). These results indicate that *bla*_GES-16_ is embedded in distinct integron-associated structures, consistent with the well-established role of class 1 integrons as platforms that capture, rearrange, and disseminate *bla*_GES_ allelic variants across bacterial hosts and ecological niches (Tavares et al., 2025).

## Conclusions

In summary, this study provides genomic evidence that WWTPs serve as key environments for AMR evolution, acting as environmental reservoirs and dissemination pathways, when treatment is inadequate, for CPE strains and MGEs linked to the spread of carbapenemase genes. The detection of these bacteria and genetic elements outside clinical settings underscores the ecological connectivity between human-associated pathogens and the environment, while also highlighting raw wastewater as an early warning signal of the circulation of high-risk lineages within the human population. Thus, the diversity of genetic platforms associated with serine carbapenemase genes in *Enterobacterales* from WWTPs highlights the dynamic nature of MGEs in driving AMR dissemination. Therefore, continuous genomic surveillance in extra-hospital compartments is essential to monitor emerging AMR genotypes and detect early signs of clinically relevant dissemination.

## Supplementary Information

Below is the link to the electronic supplementary material.

ESM 1 (DOCX 42.9 KB)

## Data Availability

The nucleotide sequences of all CPE strains used in this study are available in the NCBI database under the BioProject number PRJNA1216268.
