# Supplementary material for: Genomic characterization of carbapenemase-producing Enterobacterales from wastewater reveals the convergence of KPC-2 and GES-16 in Brazil
Source: Environ Monit Assess. 2026 Aug 31;198(9):1014. doi: 10.1007/s10661-026-15867-0 (PMC13529879; doi:10.1007/s10661-026-15867-0)
Supplement: Supplementary file 1 [file 10661_2026_15867_MOESM1_ESM.docx]

**Supplementary Material**

**Genomic characterization of carbapenemase-producing *Enterobacterales* from wastewater reveals the convergence of KPC-2 and GES-16 in Brazil**

**Authors:**

João Pedro Rueda Furlan^1,2,3,^*, Giovanna Carrasco Bueno^2,3^, Rubens Renato Sousa-Carmo^4^, Renan Lourenço Oliveira Silva^5^, Mikaela Renata Funada Barbosa^5^, Maria Ines Zanoli Sato^5^, Nilton Lincopan^3,4,6^, and Sergio Schenkman^2,3,^*

**Affiliations:**

^1^ Department of Pharmaceutical Sciences, Health Sciences Center, Federal University of Paraíba, João Pessoa, Paraíba, Brazil.

^2^ Escola Paulista de Medicina, Universidade Federal de São Paulo, São Paulo, Brazil.

^3^ Antimicrobial Resistance Institute of São Paulo (ARIES), São Paulo, Brazil.

^4^ Department of Microbiology, Instituto de Ciências Biomédicas, University of São Paulo, São Paulo, Brazil.

^5^ Department of Environmental Analysis, Environmental Company of São Paulo State (CETESB), São Paulo, Brazil.

^6^ Department of Clinical Analysis, Faculty of Pharmacy, University of São Paulo, São Paulo, Brazil.

*** Corresponding authors:**

Prof. João Pedro Rueda Furlan ([joao.furlan@academico.ufpb.br](mailto:joao.furlan@academico.ufpb.br))

Prof. Sergio Schenkman ([sschenkman@unifesp.br](mailto:sschenkman@unifesp.br))

**Supplementary Table S1.** Raw reads and genome assembly statistics for carbapenemase-producing strains

| **Strain** | **Species** | **Number of reads** | **Size (bp)^1^** | **N50 (bp)^1^** | **Genome**  **coverage (×)** | **Completeness (%)** | **Contamination (%)** | **GenBank accession number** |
| --- | --- | --- | --- | --- | --- | --- | --- | --- |
| M1 | *Enterobacter kobei* | 9,383,712 | 4935041 | 139931 | 281 | 100.00 | 0.41 | JBUYFA000000000 |
| M2 | *Klebsiella pneumoniae* | 11,048,270 | 5860616 | 158604 | 301 | 100.00 | 0.65 | JBUYEZ000000000 |
| M5 | *Klebsiella pneumoniae* | 12,089,296 | 5716193 | 164437 | 329 | 100.00 | 1.11 | JBUYEY000000000 |
| M9 | *Enterobacter asburiae* | 11,243,944 | 5092609 | 102916 | 337 | 99.89 | 0.41 | JBUYEX000000000 |
| M15 | *Klebsiella pneumoniae* | 10,243,802 | 5715810 | 179104 | 388 | 100.00 | 1.11 | JBUYEW000000000 |
| M37 | *Enterobacter kobei* | 14,421,002 | 5001019 | 197900 | 432 | 100.00 | 0.41 | JBUYEV000000000 |
| M17 | *Klebsiella pneumoniae* | 9,603,680 | 5703448 | 177634 | 261 | 99.69 | 1.11 | JBUYEU000000000 |
| M36 | *Klebsiella pneumoniae* | 14,824,064 | 5785838 | 167623 | 404 | 100.00 | 0.70 | JBUYET000000000 |
| M30 | *Klebsiella pneumoniae* | 13,118,994 | 5802114 | 165092 | 357 | 100.00 | 0.58 | JBUYES000000000 |
| M33 | *Klebsiella pneumoniae* | 13,398,934 | 5900903 | 138888 | 365 | 100.00 | 0.70 | JBUYER000000000 |
| M32 | *Klebsiella pneumoniae* | 12,468,176 | 5803044 | 165092 | 340 | 100.00 | 0.58 | JBUYEQ000000000 |
| M35 | *Klebsiella pneumoniae* | 13,581,784 | 5715984 | 181581 | 370 | 100.00 | 1.11 | JBUYEP000000000 |

^1^ bp, base pairs.

**Supplementary Table S2.** Phenotypic detection and inhibition of carbapenemases

| **Strain** | **Carbapenemase activity** | **Inhibition^1^** | | **Interpretation^2^** |
| --- | --- | --- | --- | --- |
|  |  | **EDTA** | **PBA** |  |
| M1 | Positive | Negative | Positive | Serine carbapenemase |
| M2 | Positive | Negative | Positive | Serine carbapenemase |
| M5 | Positive | Negative | Positive | Serine carbapenemase |
| M9 | Positive | Negative | Positive | Serine carbapenemase |
| M15 | Positive | Negative | Positive | Serine carbapenemase |
| M37 | Positive | Negative | Positive | Serine carbapenemase |
| M17 | Positive | Negative | Positive | Serine carbapenemase |
| M36 | Positive | Negative | Positive | Serine carbapenemase |
| M30 | Positive | Negative | Positive | Serine carbapenemase |
| M33 | Positive | Negative | Positive | Serine carbapenemase |
| M32 | Positive | Negative | Positive | Serine carbapenemase |
| M35 | Positive | Negative | Positive | Serine carbapenemase |

^1^ EDTA, ethylenediaminetetraacetic acid; PBA, phenylboronic acid. ^2^ Phenotypic inference based on inhibitor profile.

**Supplementary Table S3.** Mutations found in quinolone resistance-determining regions of fluoroquinolone-resistant *Klebsiella pneumoniae* strains

| **Strain** | **Target** | |
| --- | --- | --- |
|  | **GyrA** | **ParC** |
| M2 | S83I | S80I |
| M5 | S83I | S80I |
| M15 | S83I | S80I |
| M17 | S83I | S80I |
| M36 | S83I | S80I |
| M30 | S83I | S80I |
| M33 | S83I | S80I |
| M32 | S83I | S80I |
| M35 | S83I | S80I |

**Supplementary Table S4.** Mutations found in polymyxin resistance determinants of colistin-resistant *Klebsiella pneumoniae* strains

| **Strain** | **Target^1^** | | | | | | |
| --- | --- | --- | --- | --- | --- | --- | --- |
|  | **MgrB** | **PhoP** | **PhoQ** | **PmrD** | **PmrA** | **PmrB** | **PmrC** |
| M2 | IS*Kpn14*-ins* | ^WT^ | ^WT^ | ^WT^ | ^WT^ | T246A, R256G | C27F, I138V, O319R |
| M5 | ^WT^ | ^WT^ | E230K | T60M | A41T | L213M, T246A | I138V, O319R |
| M15 | ^WT^ | ^WT^ | E230K | T60M | A41T | L213M, T246A | I138V, O319R |
| M17 | ^WT^ | ^WT^ | E230K | T60M | A41T | L213M, T246A | I138V, O319R |
| M36 | ^WT^ | ^WT^ | ^WT^ | ^WT^ | ^WT^ | T246A, R256G | C27F, I138V, O319R |
| M32 | ^WT^ | ^WT^ | ^WT^ | ^WT^ | ^WT^ | T246A, R256G | C27F, I138V, O319R |
| M35 | ^WT^ | ^WT^ | E230K | T60M | A41T | L213M, T246A | I138V, O319R |

^1^ ^WT^, wild type. *, The insertion of ISK*pn14* at the 5′ region of mgrB truncated the start codon.

**Supplementary Table S5.** Tolerance genes to biocides and metals identified in carbapenemase-producing strains

| **Strain** | **Biocide tolerance gene^1^** | **Metal tolerance gene** |
| --- | --- | --- |
| M1 | nd | *pcoBCRSE*, *silFR* |
| M2 | *qacEΔ1* | *arsRDABC*, *pcoABCDRSE*, *silESRCFBAGP* |
| M5 | nd | *arsRDABC*, *pcoABCDRSE*, *silESRCFBAGP* |
| M9 | nd | *pcoACDRE*, *silB* |
| M15 | nd | *arsRDABC*, *pcoABCDRSE*, *silESRCFBAGP* |
| M37 | *qacL* | *arsRCR*, *merACDEPRT*, *pcoBCRSE*, *silFR* |
| M17 | nd | *arsRDABC*, *pcoABCDRSE*, *silESRCFBAGP* |
| M36 | *qacEΔ1* | *arsRDABC*, *pcoABCDRSE*, *silESRCFBAGP* |
| M30 | *qacEΔ1* | *arsRDABC*, *pcoE*, *silESRCFBAGP* |
| M33 | *qacEΔ1* | *arsRDABC*, *pcoABCDRSE*, *silESRCFBAGP* |
| M32 | *qacEΔ1* | *arsRDABC*, *pcoABCDRSE*, *silESRCFBAGP* |
| M35 | nd | *arsRDABC*, *pcoABCDRSE*, *silESRCFBAGP* |

^1^ nd, not detected.
